# Urinary mRNA Signatures as Predictors of Renal Function Decline in Patients With Biopsy-Proven Diabetic Kidney Disease

**DOI:** 10.3389/fendo.2021.774436

**Published:** 2021-11-09

**Authors:** Yu Ho Lee, Jung-Woo Seo, Miji Kim, Donghyun Tae, Junhee Seok, Yang Gyun Kim, Sang-Ho Lee, Jin Sug Kim, Hyeon Seok Hwang, Kyung-Hwan Jeong, Ju-Young Moon

**Affiliations:** ^1^ Division of Nephrology, Department of Internal Medicine, CHA Bundang Medical Center, CHA University, Seongnam, South Korea; ^2^ Division of Nephrology, Department of Internal Medicine, Kyung Hee University School of Medicine, Seoul, South Korea; ^3^ School of Electrical Engineering, Korea University, Seoul, South Korea

**Keywords:** diabetic kidney disease, mRNA, urine, biomarker, renal pathology

## Abstract

The clinical manifestations of diabetic kidney disease (DKD) are more heterogeneous than those previously reported, and these observations mandate the need for the recruitment of patients with biopsy-proven DKD in biomarker research. In this study, using the public gene expression omnibus (GEO) repository, we aimed to identify urinary mRNA biomarkers that can predict histological severity and disease progression in patients with DKD in whom the diagnosis and histologic grade has been confirmed by kidney biopsy. We identified 30 DKD-specific mRNA candidates based on the analysis of the GEO datasets. Among these, there were significant alterations in the urinary levels of 17 mRNAs in patients with DKD, compared with healthy controls. Four urinary mRNAs—*LYZ, C3, FKBP5*, and *G6PC*—reflected tubulointerstitial inflammation and fibrosis in kidney biopsy and could predict rapid progression to end-stage kidney disease independently of the baseline eGFR (tertile 1 *vs*. tertile 3; adjusted hazard ratio of 9.68 and 95% confidence interval of 2.85–32.87, *p* < 0.001). In conclusion, we demonstrated that urinary mRNA signatures have a potential to indicate the pathologic status and predict adverse renal outcomes in patients with DKD.

## Introduction

Diabetic kidney disease (DKD) is the leading cause of end-stage kidney disease (ESKD) globally, including in Korea (1). The diagnosis of DKD is traditionally based on the assessment of persistent albuminuria and decline of estimated glomerular filtration rate (eGFR); renal biopsy is not routinely performed as the natural course of DKD has previously been described as predictable (2, 3). However, it is difficult to unify the clinical spectrums of DKD as a simple and predictable disease due to the complexity of its pathogenesis and its various progression patterns (4). A large epidemiological study has revealed the decreasing prevalence of albuminuria and increasing prevalence of eGFR in DKD over the last 3 decades (5). Moreover, non-diabetic renal disease (NDRD) is frequently detected among diabetic patients who have undergone renal biopsy, raising a concern that patients with clinically diagnosed DKD may have associated NDRD (6–10). Thus, identifying patients in whom DKD diagnosis has been confirmed through kidney biopsy is an essential prerequisite for the successful discovery of relevant biomarkers. Unfortunately, this approach has rarely been used in the field of DKD research, partially justifying the reason for the validation failure of previously identified DKD biomarkers (11). Nonetheless, the incidence of biopsy-proven DKD has been increasing over the past decades (12).

The Renal Pathology Society has proposed pathologic classifications of DKD based on glomerular, tubulointerstitial, and vascular compartments (13). Several studies have consistently shown that this classification system is valuable in predicting a subsequent decline in kidney function (14–18). Nonetheless, its relevance is largely limited in clinical practice since most patients suspected to have DKD do not undergo renal biopsy. Meanwhile, non-invasive biomarkers that can reflect intrarenal pathology might be useful in predicting the renal prognosis in patients with DKD and absence of kidney biopsy. In this regard, we have previously identified that urinary CXCL16 and endostatin, indicative of the degree of tubulointerstitial fibrosis, successfully predicted poor renal outcomes in patients with biopsy-proven advanced DKD (18).

Over the past decade, omics technologies have been increasingly applied for the identification of biomarkers, including in kidney diseases (19). These web-based data platforms allow us to generate molecular profiles and assess the relevance of biological pathways, networks, potential targets, and biomarkers in diseases. In this study, through utilization of the public Gene Expression Omnibus (GEO) repository, we aimed to identify urinary mRNA biomarkers that can predict disease progression in patients with biopsy-proven DKD.

## Materials and Methods

### Patient Selection and Study Design

**Figure 1:**
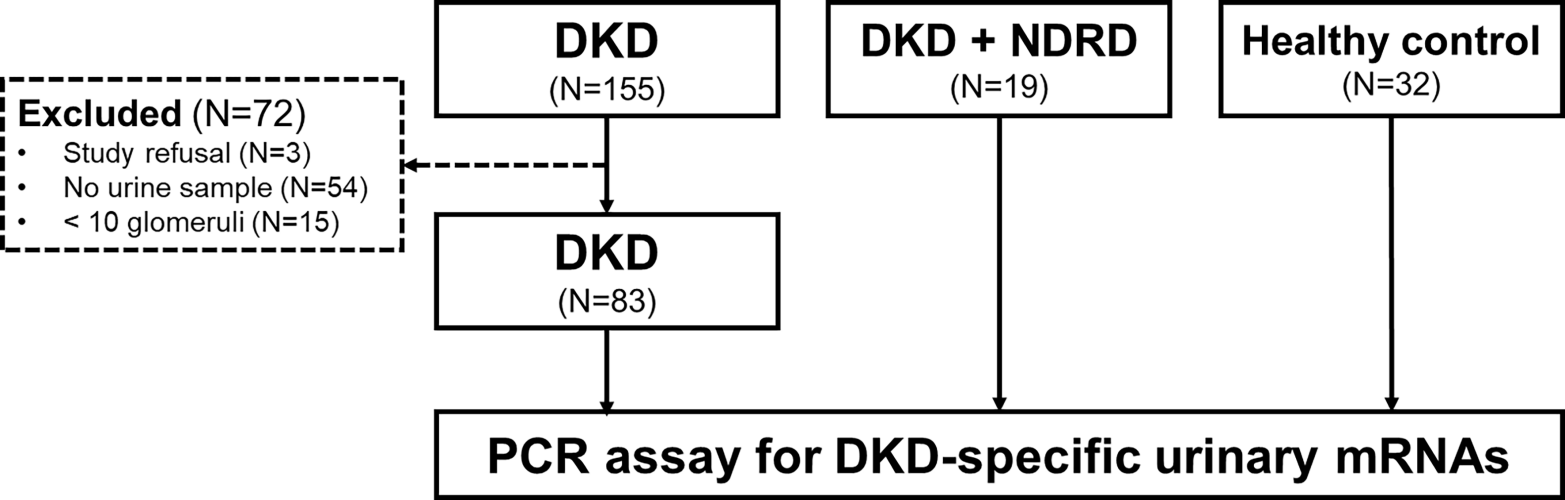
Flowchart of participant selection. We first screened 155 patients with diabetic kidney disease (DKD) whose diagnoses were confirmed by kidney biopsy. Among these, 83 patients with availability of urine samples were enrolled in this study. We also recruited 19 patients exhibiting both DKD and non-diabetic renal disease (NDRD) and 32 healthy individuals as control groups. Urinary cell pellets from the participants were collected and analyzed for measurement of the levels of DKD-specific mRNA candidates selected based on the metanalysis of the public GEO repository. DKD, diabetic kidney disease; NDRD, non-diabetic renal disease; PCR, polymerase chain reaction.

An overview of the study design and patient recruitment strategy is illustrated in **Figure 1**. We retrospectively screened 155 patients with biopsy-proven isolated DKD without NDRD at Kyung Hee Medical Center and Kyung Hee University Hospitals at Gangdong from January 2010 to March 2020. The patients were excluded in the following circumstances: unavailability of urine sample, refusal for sample collection, or biopsy samples containing <10 glomeruli. Finally, we enrolled 83 patients with DKD whose urine samples were available. We also recruited 19 patients with combined NDRD and DKD and 32 healthy controls. Individuals fulfilling all the following criteria were included as healthy controls: 1) normal renal function (eGFR > 90 ml/min/1.73 m^2^), 2) absence of proteinuria or hematuria, and 3) absence of diabetes or hypertension. Indications for renal biopsy in diabetic patients are described elsewhere (6).

The baseline characteristics and laboratory parameters of the enrolled patients were collected at the time of renal biopsy. Renal function was assessed by eGFR, calculated using the Chronic Kidney Disease Epidemiology Collaboration formula (20). Renal outcomes were defined as progression to ESKD requiring renal replacement therapy or transplantation.

### Ethics Statement

This study was conducted according to The Code of Ethics of the World Medical Association (Declaration of Helsinki), and was reviewed and approved by the local ethics committee (IRB no. KHNMC2021-01-054-003). Informed consent was obtained from the study participants.

### Pathologic Diagnoses of Diabetic Kidney Diseases and Non-Diabetic Renal Disease

All biopsy specimens were processed by standard methods and routinely examined by light microscopy, immunofluorescence, and electron microscopy. The diagnosis of DKD was made and categorized according to the pathologic classification of the Renal Pathology Society (13). In brief, this classification system includes five histologic parameters: glomerular classification, interstitial fibrosis and tubular atrophy (IFTA), interstitial inflammation, arterial hyalinosis, and arteriosclerosis. The diagnosis of NDRD accompanied with DKD was made when the kidney biopsy tissue exhibited typical features of both DKD and other glomerulopathies.

### Selection of Diabetic Kidney Disease-Specific mRNA Candidates

Upon searching through the GEO database using the keywords “diabetic kidney disease” and “diabetic nephropathy,” we found two data sets (GSE104948 and GSE104954) with the whole gene expression profiles of both DKD patients and corresponding healthy controls. The meta-analysis of the two data sets was performed by GeneMeta R package that follows the approach of Choi et al. (21). Random effects models were used for the meta-analysis. The false discovery rates (FDRs) were obtained from 1,000 permutations, and the effective fold changes were calculated as the average fold changes of two data sets weighted by the number of samples. Those with fold change ≥2 or ≤0.5, and FDR <0.001 were selected as the mRNA candidates in each data set.

### Collection of Urinary Samples and Measurements of Urinary mRNA Levels

Urine sample collection, processing, and storage was performed in an aseptic manner by an experienced technician to avoid cross-contamination. Mid-stream urine samples were collected on the day of renal biopsy or at the time of visit for medical checkup and were centrifuged at 2,000 g for 20 min at room temperature. Cell pellets were separated on clean benches, subsequently transferred into RNA (Invitrogen, Carlsbad, CA), and stored at -80°C until required. All these processes were performed immediately after urine sample collection; therefore, the urine samples were stored within 1 hour of collection. Total RNA was extracted using the PureLinkTM RNA Mini Kit (Invitrogen), according to the manufacturer’s recommendations. The amount of total RNA (ug) was measured using a NanoDrop^®^ ND-2000 UV spectrophotometer (Thermo Scientific, Waltham, MA), cDNA synthesis was performed with the total RNA using M-MLV RT enzyme (200 U/µl; Mbiotech, Inc., Seoul, Korea), and the levels of gene expressions using each target primer and SYBR Green Master Mix (Applied Biosystems, Foster city, CA) were measured on ABI StepOne real-time polymerase chain reaction system (Applied Biosystems), as previously described (22). Each mRNA level was normalized by 18S rRNA used as an endogenous control for the 2-ΔΔCt method, and then log_10_-transformed to reduce deviation.

### Statistical Analyses

All statistical analyses were performed with SPSS for Windows, version 20.0 (IBM, Armonk, NY). Baseline characteristics and clinical parameters are expressed as the mean ± standard deviation or as number of patients and percentage. Analysis of variance and Bonferroni post-hoc test was used for comparisons of urinary mRNA levels among different groups. The combined scores of mRNA signatures were determined by calculating the predicted probabilities of ESKD progression for each patient using logistic regression analysis. Patients were then divided into tertiles according to their values of calculated probability. Kaplan–Meier curves were generated to illustrate the cumulative probabilities of renal outcomes, and the Cox proportional hazards model was used for the multivariable analysis.

## Results

### Baseline Clinical Parameters and Pathologic Features of Enrolled Patients

Baseline demographics of the patients with DKD are shown in **Table 1**. The mean age was 55.2 years, 63.9% (53/83) were male, and the mean duration of diabetes was 11.3 years. Most patients exhibited moderate-to-severe renal dysfunction, with a mean eGFR of 45.5 mL/min/1.73 m^2^ and a mean urinary protein-to-creatinine ratio of 6.0 g/gCr. During the 2.6 years of mean follow-up period, death-censored renal outcomes occurred in 35 (42.2%) of the patients. Healthy controls were significantly younger, whereas patients with combined NDRD and DKD were older, compared to those with DKD alone (*p*<0.001 and *p*=0.020, respectively; **Supplementary Table 1**). Baseline renal function and the amount of proteinuria were comparable between patients with DKD and those with combined NDRD and DKD.

**Table 1:** Baseline characteristics and clinical parameters of patients with diabetic kidney disease.

Number of patients	83
Age (year)	55.4±10.6
Sex (Male, %)	53 (63.9)
Body mass index (kg/m^2^)	25.1±3.0
Duration of diabetes (years)	11.3±8.1
Presence of diabetic retinopathy (n, %)	59/80 (71.7)^a^
Hypertension (n, %)	67 (80.7)
HbA1c (%)	7.9±2.0
Hemoglobin (g/dL)	10.7±2.1
eGFR (ml/min/1.73m^2^)	45.5±30.3
Albumin (g/dL)	3.2±0.6
Urine protein-to-creatinine ratio (g/gCr)	6.0±4.2
Death-censored ESKD progression (n, %)	35 (42.2)

Values are expressed as mean ± standard deviation or number of patients (percentage).

a Not assessed in three patients.

eGFR, estimated glomerular filtration rate; ESKD, end stage kidney disease.

Histologic examination revealed that 75.9% (63/83) of patients with DKD showed advanced glomerular injuries (36 [43.4%] and 27 [32.5%] for glomerulonephritis class III and IV, respectively; **Table 2**). Advanced tubulointerstitial fibrosis was observed in 30.1% of these patients (19 [22.9%] and 6 [7.2%] for IFTA scores of 2 and 3, respectively). Interstitial inflammation was also frequently observed, either in relation to IFTA or in areas without IFTA (44 [53.0%] and 27 [32.5%] for interstitial inflammation score of 1 and 2, respectively).

**Table 2:** Pathologic classifications of patients with diabetic kidney disease.

Glomerular classification	
Class II	20 (24.1)
Class III	36 (43.4)
Class IV	27 (32.5)
IFTA	
0	4 (4.8)
1	54 (65.1)
2	19 (22.9)
3	6 (7.2)
Interstitial inflammation	
0	12 (14.5)
1	44 (53.0)
2	27 (32.5)
Arterial hyalinosis	
0	9 (10.8)
1	56 (67.5)
2	18 (21.7)
arteriosclerosis	
0	16 (19.3)
1	51 (61.4)
2	16 (19.3)

IFTA, interstitial inflammation and tubular atrophy.

### Identification of Diabetic Kidney Disease-Specific mRNA Candidates Using GEO Database

From the GEO database, we found two datasets that contained transcriptomic profiles of kidney tissues obtained from 14 DKD patients and 36 healthy kidney donors. A meta-analysis was performed using the two datasets to find the relevant genes in which the expression patterns were significantly different between the groups. Among 150 genes with the lowest false discovery rate, we selected the top 20 up-regulated and 10 down-regulated genes in DKD tissues in the order of the fold changes (**Table 3**).

**Table 3:** List of diabetic kidney disease-specific urinary mRNA candidates identified by GEO dataset analysis.

Upregulated in DKD		Down-regulated in DKD	
Genes	Fold change	Genes	Fold change
*LYZ*	6.55	*APOLD1*	0.38
*CX3CR1*	4.71	*FABP1*	0.36
*WFDC2*	4.21	*HPD*	0.36
*NNMT*	4.01	*CTSV*	0.36
*C3*	3.72	*LPL*	0.32
*MEST*	3.57	*G6PC*	0.29
*THBS2*	3.39	*FKBP5*	0.27
*MOXD1*	3.09	*ZBTB16*	0.27
*CLU*	2.90	*PDK4*	0.23
*HOPX*	2.87	*CYP27B1*	0.22
*COL3A1*	2.86		
*PLK2*	2.84		
*EVI2A*	2.75		
*TNFAIP8*	2.65		
*LY96*	2.62		
*COMP*	2.51		
*SPON2*	2.49		
*CFB*	2.47		
*SOX4*	2.41		
*COL1A1*	2.39		

DKD, diabetic kidney disease.

### Urinary Levels of DKD-Specific mRNA Candidates in Different Diagnostic Groups

**Figure 2:**
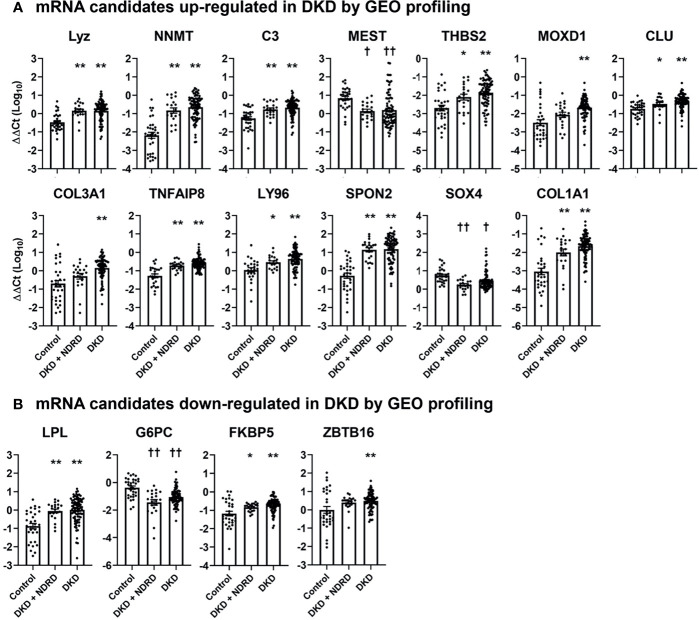
Urinary levels of diabetic kidney disease-specific mRNA candidates in healthy controls, patients with combined diabetic kidney disease and non-diabetic renal disease, and those with isolated diabetic kidney disease. The levels of selected mRNA biomarker candidates whose expressions are significantly altered between the different groups are shown. **(A, B)** mRNA candidates up-regulated **(A)** and down-regulated **(B)** in DKD *via* GEO profiling. mRNA levels are measured by quantitative real-time polymerase chain reaction and are expressed as log-transformed delta-delta cycle threshold (ΔΔCt) after an adjustment by 18S rRNA and controls. Five mRNAs among those listed in (*CX3CR1, HOPX, COMP, APOLD1*, and *CYP27B*) are not illustrated in this figure as these mRNAs failed to pass the quality control process. ^*^ *p* < 0.05, ^**^ *p* < 0.005, up-regulated *vs*. control; ^†^ *p* < 0.05, ^††^ *p* < 0.005, down-regulated *vs*. control. DKD, diabetic kidney diseases; NDRD, non-diabetic renal disease; GEO, gene expression omnibus.

We next measured the levels of each mRNA candidate in the urine of healthy controls, patients with combined DKD and NDRD, and those with DKD alone. Five mRNAs failed to pass the quality control process (i.e., undetectable mRNA levels in >20% of samples) and were excluded from the analysis. Among the 17 up-regulated and 8 down-regulated mRNA candidates, 13 (76.5%) and 4 (50.0%) genes showed significantly altered expressions in the urine of patients with DKD compared to those of healthy controls, respectively (**Figure 2**). Most DKD-specific mRNA candidates up-regulated in GEO profiling were actually increased (84.6%, 11/13). In contrast, 75% of mRNAs (3/4) down-regulated in GEO profiling were paradoxically increased in the urine of patients with DKD. Notably, the expression profiles of the urinary mRNAs in patients with combined DKD and NDRD were substantially similar to those in patients with DKD alone.

### Levels of Urinary mRNAs According to Pathologic Classification of Diabetic Kidney Disease

**Figure 3:**
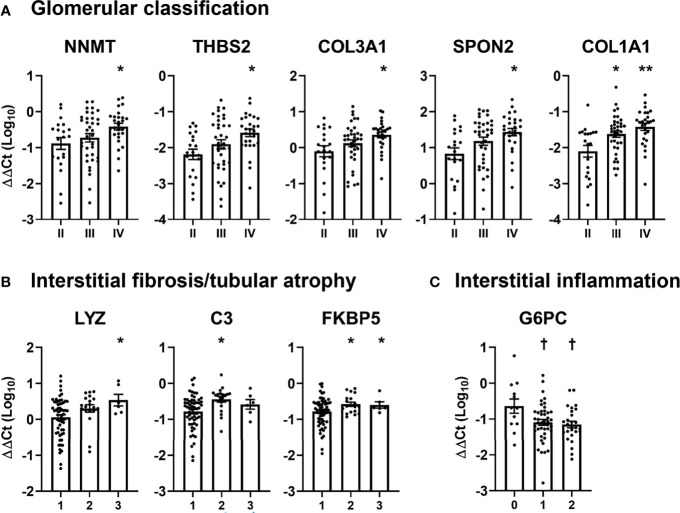
Association between pathologic classifications and urinary mRNA levels in patients with diabetic kidney disease. **(A–C)** The levels of significantly altered urinary mRNAs according to **(A)** glomerular classification, **(B)** interstitial fibrosis and tubular atrophy, and **(C)** interstitial inflammation scores in patients with diabetic kidney disease are shown. Levels of each mRNA are expressed as log-transformed delta-delta cycle threshold (ΔΔCt) after adjusting for 18S rRNA and controls. ^*^ *p* < 0.05, ^**^ *p* < 0.005, up-regulated *vs*. glomerular class II or IFTA score of 1; ^†^ *p* < 0.05, down-regulated *vs*. interstitial inflammation score of 0.

Subsequently, we examined the relationship between DKD-specific mRNAs and pathologic classification of DKD (**Figure 3**). Patients with glomerulonephritis class IV showed significantly higher urinary levels of five mRNAs (nicotinamide N-methyltransferase [*NNMT*], thrombospondin 2 [*THBS2*], collagen type III alpha 1 chain [*COL3A1*], spondin 2 [*SPON2*], and collagen type I alpha 1 chain [*COL1A1*]), compared with those exhibiting glomerulonephritis class II (**Figure 3A**). Meanwhile, three mRNAs (lysozyme [*LYZ*], complement 3 [*C3*], and FK506 binding protein 5 [*FKBP5*]) were positively associated with the IFTA score, while one mRNA (glucose-6-phosphatase [*G6PC*]) was negatively associated with the degree of interstitial inflammation (**Figures 3B, C**). No mRNA showed a significant relationship with the severity of arterial hyalinosis and arteriosclerosis.

### Renal Outcomes According to the Clinicopathologic Features

**Figure 4:**
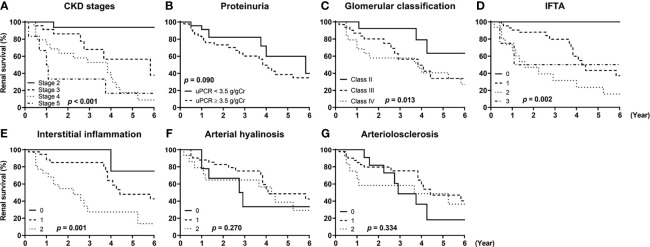
Renal survival of patients with diabetic kidney disease according to their clinicopathologic features. The renal survival of patients with diabetic kidney disease according to **(A)** stages of chronic kidney disease (CKD), **(B)** the amount of proteinuria, **(C)** glomerulonephritis classification score, **(D)** interstitial fibrosis and tubular atrophy (IFTA) score, **(E)** interstitial inflammation score, **(F)** arterial hyalinosis score, and **(G)** arteriolosclerosis score are shown. *P*-values were calculated by log-rank test. CKD, chronic kidney disease; uPCR, urinary protein-to-creatinine ratio; IFTA, interstitial fibrosis and tubular atrophy.

**Figure 4** shows the unadjusted Kaplan–Meier survival curves of the patients according to the stages of chronic kidney disease (CKD), amount of proteinuria, and the five different pathologic classifications. Advanced CKD stages were significantly associated with increased risks of ESKD progression, and the patients exhibiting nephrotic range proteinuria showed a trend for worse renal outcomes compared with those exhibiting non-nephrotic range proteinuria (**Figures 4A, B**). We also observed that glomerulonephritis classification, IFTA, and interstitial fibrosis were significantly associated with adverse renal outcomes (**Figures 4C–E**). Arterial hyalinosis and arteriolosclerosis were not predictive of ESKD progression (**Figures 4F, G**).

### Renal Outcomes According to the Levels of Compartmental mRNA Signatures

**Figure 5:**
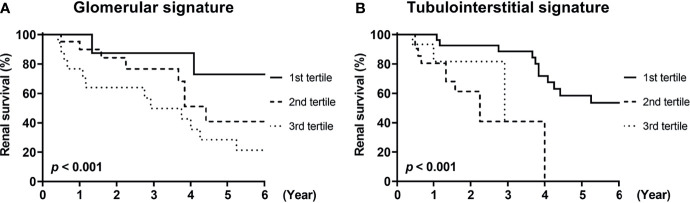
Renal survival of patients with diabetic kidney disease according to compartmental mRNA signatures. **(A, B)** The renal survival of patients with diabetic kidney disease according to the tertiles of **(A)** glomerular and **(B)** tubulointerstitial mRNA signatures are shown. Each signature was generated from the integration of mRNAs differentially expressed in corresponding compartments (*NNMT, THBS2, SPON2, COL3A1, COL1A1* for glomerular signature and *LYZ, C3, FKBP5, G6PC* for tubulointerstitial signature). *p* < 0.001 for both comparisons by log-rank test.

Finally, we investigated whether urinary mRNAs can be used as the predictor of renal outcomes in patients with DKD. To this end, mRNAs associated with glomerular and tubulointerstitial injuries were integrated to generate gene signatures of each compartment. The cumulative incidence of renal outcomes was significantly increased in patients with third tertiles of glomerular or tubulointerstitial mRNA signatures (*p*<0.001 for both comparisons; **Figure 5**). Univariate Cox regression analysis consistently demonstrated that patients in the third tertiles of glomerular and tubulointerstitial mRNA signatures showed significantly higher risk of ESKD progression than those in the first tertiles (**Table 4**). Interestingly, the significant associations between glomerular mRNA signatures and renal outcomes disappeared when baseline renal function was added as an adjustment variable (hazard ratios [HR] of 1.80, 95% confidence interval [CI] of 0.46–7.06, *p*=0.402). In contrast, tubulointerstitial mRNA signatures maintained their significant associations with poor renal outcomes even after the adjustments with baseline renal function (HR of 9.68, 95% CI of 2.85–32.87, *p*<0.001).

**Table 4:** Hazard ratios of compartmental mRNA signatures for renal survival.

		Unadjusted		Model 1^a^		Model 2^b^	
		HR (95% CI)	*p* value	HR (95% CI)	*p* value	HR (95% CI)	*p* value
Glomerular signatures^c^	Tertile 1	Reference	–	Reference	–	Reference	–
	Tertile 2	2.62 (0.82 – 8.40)	0.106	2.82 (0.84 – 9.47)	0.093	1.61 (0.43 – 5.96)	0.477
	Tertile 3	6.50 (2.22 – 19.08)	0.001	6.07 (1.93 – 19.06)	0.002	1.80 (0.46 – 7.06)	0.402
Tubulointerstitial signatures^d^	Tertile 1	Reference	–	Reference	–	Reference	–
	Tertile 2	6.93 (2.35 – 20.40)	<0.001	11.47 (3.27 – 40.24)	<0.001	7.77 (2.51 – 23.68)	<0.001
	Tertile 3	7.62 (2.24 – 25.92)	0.001	11.73 (3.07 – 44.87)	<0.001	9.68 (2.85 – 32.87)	<0.001

a Model 1: adjusted for age, sex, hypertension, and urinary protein-to-creatinine rate.

b Model 2: model 1 + estimated glomerular filtration rate.

c Composed of urinary NNMT, THBS2, SPON2, COL3A1, and COL1A1 mRNA levels.

d Composed of urinary FKBP5, C3, LYZ, and G6PC mRNA levels.

HR, hazard ratio; CI, confidence interval.

## Discussion

In this study, we analyzed the clinicopathologic data and various urinary mRNAs to discover novel, non-invasive biomarkers that could predict renal outcomes in patients with biopsy-proven DKD. Utilizing public GEO datasets, we extracted 30 mRNAs as biomarker candidates; we observed that levels of 17 mRNAs were significantly altered in the urine of patients with DKD, compared to those of healthy controls. Among these, five and four mRNAs showed significant associations with the pathologic severity of glomerular and tubulointerstitial compartments, respectively. Finally, four urinary mRNAs—*LYZ, C3, FKBP5*, and *G6PC*—were observed to be associated with tubulointerstitial injury and could predict DKD progression independently from baseline clinical parameters, including residual kidney functions. Together, these data suggest that urinary tubulointerstitial mRNA signatures may help identify those at high risk of progression to ESKD.

Urine is a valuable source for identifying relevant biomarkers associated with kidney diseases as it is generated directly from the kidneys and can be collected non-invasively. We have previously demonstrated the utility of urinary mRNAs and proteins as diagnostic and prognostic biomarkers in various renal conditions such as transplant rejection, primary glomerular diseases, and DKD (18, 22–28). Recent advances in the utilization of open data resources have further enhanced the potentials of urinary mRNAs in identifying biomarkers. Using open datasets of DKD and applying an integrative bioinformatics approach, Zhou et al. revealed urinary *BBOX1* to be a non-invasive diagnostic biomarker of DKD in diabetic patients who did not undergo kidney biopsy (29). In this study, we were able to eliminate the possibility of the presence of unexpected NDRD and determine the relationship between renal histology and urinary mRNAs by including patients whose diagnosis was confirmed by renal biopsy, emphasizing the importance of pathologic data in a DKD study.

The clinical significance of pathologic classifications of DKD in predicting renal outcomes has been consistently demonstrated in previous studies (14–18), supporting the idea that urinary mRNAs reflecting intrarenal pathology could be prognostic biomarkers in patients with DKD. In this study, we observed that several DKD-specific urinary mRNAs were significantly associated with the severity of pathologic findings in the kidneys as well as renal outcomes (**Figures 3** and **5**). Although the pathophysiologic roles of selected mRNAs were not investigated here, previous studies have shown glomerular compartmental mRNAs, comprising *NNMT, THBS2, SPON2, COL3A1*, and *COL1A1*, to be involved in podocyte damage (30–32) and glomerulosclerosis (33, 34), and tubulointerstitial compartmental mRNAs, comprising *LYZ, C3, FKBP5*, and *G6PC*, to be associated with fibrosis (35–37) and inflammation (38). Notably, those mRNAs reflected different compartments of the kidneys in an exclusive manner, suggesting that glomerular and tubulointerstitial injuries might result in discriminative urinary mRNA expressions. In line with our data, a recent study performed transcriptomic analysis of micro-dissected kidneys and showed discriminative gene expression patterns between glomerular and tubulointerstitial compartments (39).

Among the differentially expressed mRNAs, those up-regulated in the patients with DKD were predominantly involved in immune response and inflammation (*CLU, C3, CFB, LY96, SPON2, CX3CR1, FKBP5, TNFAIP8*), and extracellular matrix organization (*COMP, COL1A1, COL3A1, THBS2, SPON2, MOXD1*); those down-regulated in the patients with DKD were mainly associated with metabolic pathways (*APOLD1, FABP1, HPD, LPL, G6PC, PDK4*). The overall trends were consistent with those reported in previous studies that have investigated transcriptomic profiles of renal tissues obtained from advanced human diabetic nephropathy (39, 40). Notably, most mRNAs (11/13, 84.6%) among those up-regulated in patients with DKD *via* GEO profiling showed increased levels in the urine. In contrast, only one mRNA (1/4, 25%) among those down-regulated in patients with DKD *via* GEO profiling showed decreased levels in the urine (**Figures 2A** and **1B**). Although the reasons for this discrepancy could not be identified in this study, the mRNA expression profiles of the cells might have been altered once they were detached from the kidneys and released into the urine.

Our data suggest that urinary mRNAs may be potential predictors of renal function decline in patients with advanced DKD. In particular, mRNA signatures of tubulointerstitial inflammation and fibrosis were a significant predictor of poor renal outcomes even after multivariable adjustments, including baseline renal function. In contrast, the predictive power of glomerular mRNA signatures in predicting renal outcomes was lost after adjustments for eGFR. These results suggest that tubulointerstitial mRNA signatures may be potential independent predictors of rapid decline in renal function, whereas glomerular mRNA signatures are not. Similarly, in line with the findings of previous studies, we revealed the advantages of tubulointerstitial injury scores over glomerular classifications in the prediction of renal outcomes among patients with DKD exhibiting advanced glomerular injuries (16, 18).

Normalization of urinary mRNA expression data is a critical issue in biomarker research; however, optimal normalization strategies for mRNA remain controversial (41). In this study, we used 18S rRNA rather than urine creatinine for the normalization of urinary mRNAs expression data as we have previously demonstrated this strategy to be useful in identifying urinary mRNA biomarkers (22, 28). Further investigations are required to determine whether urine creatinine may be better for normalization of urinary mRNA expression data.

The limitations of this study should be mentioned. We did not determine whether the mRNA signatures developed in this study could be applied to patients with early-stage DKD. Patients with early-stage DKD were not included in this study as they rarely undergo renal biopsy in clinical practice. Given that early and advanced diabetic nephropathy shows substantially different transcriptomic profiles (40), biomarkers of advanced DKD may not be useful in the early stages of DKD. In addition, DKD-specific urinary mRNA profiles could not discriminate between patients with DKD and those with combined DKD and NDRD. A possible reason for this may be that the patients in both groups had a substantial duration of diabetes (mean duration >10 years); therefore, the effects of NDRD on urinary mRNA levels were relatively insignificant compared to those of DKD. The smaller number of patients in the NDRD group as well as their diagnostic heterogeneity might have also affected these results.

In conclusion, we developed urinary mRNA signatures as predictors of rapid disease progression in patients with advanced DKD. Future prospective studies are required to confirm whether our mRNA signatures can identify those at high risk of renal function decline in a non-invasive manner.

## Data Availability Statement

The datasets presented in this study can be found in online repositories. The names of the repository/repositories and accession number(s) can be found in the article/**Supplementary Material**.

## Ethics Statement

The studies involving human participants were reviewed and approved by IRB no. KHNMC2021-01-054-003. The patients/participants provided their written informed consent to participate in this study.

## Author Contributions

Research idea and study design: YL, J-WS, and J-YM. Data acquisition: YK, S-HL, JSK, HH, and K-HJ. Data analysis/interpretation: YL, J-WS, DT, and JSK. Statistical analysis: YL, J-WS, DT, and JSK. Supervision or mentorship: J-YM. Each author contributed important intellectual content during manuscript drafting and approved the final article.

## Funding

This research was supported by Basic Science Research Program through the National Research Foundation of Korea (NRF) funded by the Ministry of Education (2018R1D1A1B05049016 and 2021R1G1A1014115).

## Conflict of Interest

The authors declare that the research was conducted in the absence of any commercial or financial relationships that could be construed as a potential conflict of interest.

## Publisher’s Note

All claims expressed in this article are solely those of the authors and do not necessarily represent those of their affiliated organizations, or those of the publisher, the editors and the reviewers. Any product that may be evaluated in this article, or claim that may be made by its manufacturer, is not guaranteed or endorsed by the publisher.
