# Supplementary material for: Urinary mRNA Signatures as Predictors of Renal Function Decline in Patients With Biopsy-Proven Diabetic Kidney Disease
Source: Front Endocrinol (Lausanne). 2021 Nov 9;12:774436. doi: 10.3389/fendo.2021.774436 (PMC8630698; doi:10.3389/fendo.2021.774436)
Supplement: Supplementary file 1 [file DataSheet_1.docx]

**Supplementary Table 1. Baseline characteristics and clinical parameters of healthy control and patients diagnosed with combined diabetic kidney diseases and non-diabetic renal diseases**

|  | Healthy control  (n=32) | DKD + NDRD  (n=19) |
| --- | --- | --- |
| Age (years) | 33.2±6.3 | 61.7±10.3 |
| Sex (Male, %) | 13 (40.6) | 12 (63.2) |
| Body mass index (kg/m^2^) | - | 25.4±4.2 |
| Duration of diabetes (years) | - | 11.4±8.8 |
| Presence of diabetic retinopathy (n, %) | - | 15/18 (81.2) ^a^ |
| Hypertension (n, %) | 0 (0) | 18 (94.7) |
| HbA1c (%) | - | 6.4±1.3 |
| Hemoglobin (g/dL) | - | 10.6±2.5 |
| eGFR (ml/min/1.73m^2^) |  | 36.2±35.5 |
| Albumin (g/dL) | - | 3.0±0.5 |
| Urine protein-to-creatinine ratio (g/gCr) | - | 9.0±10.2 |
| Types of non-diabetic renal diseases (n, %)  IgA nephropathy  Membranous nephropathy  Thrombotic microangiopathy  Membranoproliferative glomerulonephritis  Focal segmental glomerulosclerosis  Post-streptococcal glomerulonephritis  Minimal change disease  Myeloma cast nephropathy |  | 7 (36.8)  4 (21.1)  2 (10.5)  2 (10.5)  1 (5.3)  1 (5.3)  1 (5.3)  1 (5.3) |

Values are expressed as mean ± standard deviation or number of patients (percentage).

^a^ The presence of diabetic retinopathy was not assessed in a patient.

**Abbreviation**: DKD, diabetic kidney diseases; NDRD, non-diabetic renal diseases; eGFR, estimated glomerular filtration rate.
